# The complete chloroplast genome sequence of tung tree (*Vernicia fordii*): Organization and phylogenetic relationships with other angiosperms

**DOI:** 10.1038/s41598-017-02076-6

**Published:** 2017-05-12

**Authors:** Ze Li, Hongxu Long, Lin Zhang, Zhiming Liu, Heping Cao, Mingwang Shi, Xiaofeng Tan

**Affiliations:** 1 Key Laboratory of Cultivation and Protection for Non-Wood Forest Trees, Ministry of Education, Central South University of Forestry and Technology, Changsha, Hunan 410004 China; 2 Cooperative Innovation Center of Cultivation and Utilization for Non-Wood Forest Trees of Hunan Province, Central South University of Forestry and Technology, Changsha, Hunan 410004 China; 3 Department of Biology, Eastern New Mexico University, Portales, New Mexico 88130 USA; 4 U.S. Department of Agriculture, Agricultural Research Service, Southern Regional Research Center, New Orleans, Louisiana 70124 USA; 5 Henan Institute of Science and Technology, Xinxiang, Henan 453003 China

## Abstract

Tung tree (*Vernicia fordii*) is an economically important tree widely cultivated for industrial oil production in China. To better understand the molecular basis of tung tree chloroplasts, we sequenced and characterized its genome using PacBio RS II sequencing platforms. The chloroplast genome was sequenced with 161,528 bp in length, composed with one pair of inverted repeats (IRs) of 26,819 bp, which were separated by one small single copy (SSC; 18,758 bp) and one large single copy (LSC; 89,132 bp). The genome contains 114 genes, coding for 81 protein, four ribosomal RNAs and 29 transfer RNAs. An expansion with integration of an additional *rps19* gene in the IR regions was identified. Compared to the chloroplast genome of *Jatropha curcas*, a species from the same family, the tung tree chloroplast genome is distinct with 85 single nucleotide polymorphisms (SNPs) and 82 indels. Phylogenetic analysis suggests that *V. fordii* is a sister species with *J. curcas* within the Eurosids I. The nucleotide sequence provides vital molecular information for understanding the biology of this important oil tree.

## Introduction

Tung tree (*Vernicia fordii*) belongs to the Euphorbiaceae family of woody angiosperms and has been cultivated for more than 1,000 years in China. Along with oil-tea tree (*Camellia oleifera*), walnut (*Juglans regia*) and Chinese tallow tree (*Sapium sebiferum*), tung tree is considered as one of the four major woody oil trees in China^1, 2^. Tung tree grows fast, blossoms and yields fruits in three years due to its high efficiency of photosynthesis. Tung oil extracted from seed kernels containing 80% eleostearic acid, which is active for chemical polymerization^1^, and can be used as an ingredient in painting, varnishing, and other coating for enhancing adhesion and resistance to acid, alkali, frost and chemicals^3^. In recent years, tung oil has been shown with a potential for biodiesel production because tung tree grows fast with high oil yields^1, 4^. One approach to improve tung oil production would be to engineer chloroplasts with more efficient photosynthesis in tung tree leaves. Sequencing the complete chloroplast genome would facilitate the chloroplast transformation technique because the transformation of chloroplast genome has many advantages than nuclear transformation including a high level of transgene expression, lacking of gene silencing or positional effect and transgene containment^5–7^.

Chloroplast (cp) is a special subcellular organelle which contains the entire enzymatic machinery for photosynthesis and provides essential energy for green plants^8–10^. Chloroplast contains its own small genome, which usually consists of a circular double-stranded DNA molecule^10, 11^. In angiosperms, cp genomes are 120–217 kb in length^12, 13^. Most of the cp genomes contain 110–130 distinct genes, approximately 80 genes coding for proteins involved in gene expression or photosynthesis^10, 14^ and other genes coding for four rRNAs and 30 tRNAs^15, 16^. In addition, most cp genomes have four distinct regions, including a pair of inverted repeats (IRs, 20–28 kb), which are separated by a small single copy (SSC, 16–27 kb) region and a large single copy (LSC, 80–90 kb) region^14, 17, 18^. The cp genome can be used to investigate molecular evolution and phylogenies^14, 19^. Moreover, cp genomes are maternally inherited, which is beneficial in genetic engineering due to lack of cross-recombination^20, 21^.

In this study, we determined the complete sequence of the chloroplast genome of tung tree using the PacBio RS II platform. Additionally, we compared it with other known cp genomes aiming to determine phylogenetic relationships among angiosperms.

## Results

### Genome sequencing, assembly and validation

Using the third-generation sequencing (PacBio RS II System), 18.26 Gb of raw sequence data was generated from tung tree cp genome through 2,910,237 reads with a mean read length of 6,273 bp. The sequence data that satisfied the quality control standards after filtering, were used to construct the cp genome by comparing with the reference cp genomes of other 908 species in NCBI plastid database. The longest recovered subread was 35,889 bp in length and the total amount of recovered subreads was 334 Mb. The depth of average genome coverage of the subreads exceeded 2000X, suggesting that the sequencing data was sufficient to meet the assembly requirements for cp genome. Finally, we obtained 2.4 M high quality reads with a mean read length of 6,762 bp and an N50 contig size of 17,719 bp. The results showed a high consensus of the sequences except 10 different bases between IRa and IRb regions. To ensure the accuracy for the tung tree cp genome, we compared the Sanger results with the assembled genome. The sequence of tung tree cp genome has been deposited in public databases (Genbank accession number: KY628420).

### General features of tung tree cp genome

**Figure 1:**
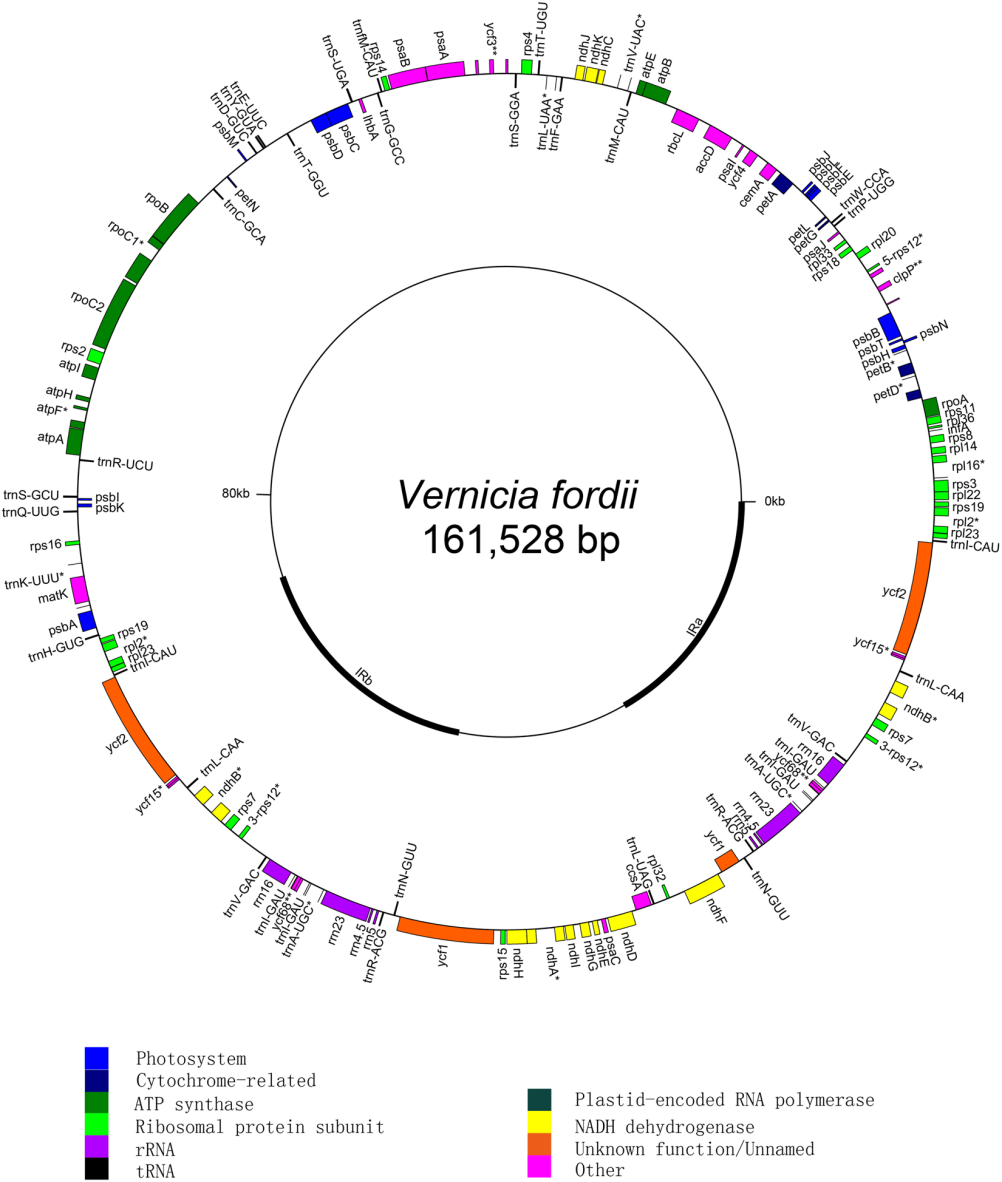
Gene map of tung tree chloroplast genome from PacBio RS II platform. The thick lines indicate the inverted repeats (IRa and IRb) which separate the genome into large single copy (LSC) and small single copy (SSC) regions. Genes shown in the inner side of the circle are transcribed clockwise, and those located on the outside of the circle are transcribed counter-clockwise.

The total length of tung tree cp genome was determined to be 161,528 bp with the circular quadripartite structure similar to major angiosperms cp genomes. The cp genome contains a small single-copy (SSC) region of 18,758 bp and a large single-copy (LSC) region of 89,132 bp, separated by two copies of an inverted repeat (IR) of 26,819 bp (Fig. 1, Table 1). The genome is structured with 114 unique genes including 81 distinct protein-coding genes, four distinct rRNA genes and 29 distinct tRNA genes (Table 2). Seven tRNA genes and all of the rRNA genes are duplicated in the IR regions, making a total number of 135 genes (Tables 1 and 2). The genes coding for proteins, rRNA, tRNA, introns, and intergenic spacers (IGSs) are 82,034, 9048, 2742, 17,821, and 52,599 bp, which represent 50.79, 5.60, 1.70, 11.03, and 32.56% of the cp genome, respectively (Table 1). In this cp genome, 16 genes including 5 tRNA genes contain introns structure (Table 2).

**Table 1:** Characteristics of tung tree plastome genome.

Sequence region	Length (bp)/Percent (%)
Total cp genome	161,528 (100.00)
LSC	89,132 (55.18)
SSC	18,758 (11.61)
IR	26,819 (16.60)
Coding regions	91,388 (57.20)
Protein-coding regions	82,034 (50.79)
Introns	17,821 (11.03)
rRNA	9,048 (5.60)
tRNA	2,742 (1.70)
IGS	52,599 (32.56)
**GC content**	**Length (bp)/Percent (%)**
Overall GC size	58,188 (36.02)
Overall A size	52,378 (32.43)
Overall T size	50,962 (31.55)
Overall G size	29,615 (18.33)
Overall C size	28,573 (17.69)
GC content in protein-coding regions	30,780 (37.52)
GC content in IGS	15,394 (29.27)
GC content in introns	6,595 (37.01)
GC content in tRNA	2,742 (53.17)
GC content in rRNA	5,014 (55.42)
**Gene classification**	**Number**
Total genes	135
Protein-coding genes	81
rRNA genes	4
tRNA genes	29
Genes with introns	16
Genes duplicated by IR	21

**Table 2:** Genes locating on tung tree cp genome.

Gene categories	Groups of genes	Name of genes
Genes for photosynthesis	Subunits of photosystem I	*psaA, psaB, psaC, psaI, psaJ*
	Subunits of photosystem II	*psbA, psbB, psbC, psbD, psbE, psbF, psbH, psbI, psbJ, psbK, psbL, psbM, psbN, psbT*
	Subunits of ATP synthase	*atpA, atpB, atpE, atpF* ^b^ *, atpH, atpI*
	Subunits of cytochrome b/f complex	*petA, petB* ^b^ *, petD* ^b^ *, petG, petL, petN*
	Subunits of NADH-dehydrogenase	*ndhA* ^b^ *, ndhB* ^a,b^ *, ndhC, ndhD, ndhE, ndhF, ndhG, ndhH, ndhI, ndhJ, ndhK*
	Large subunit of RuBisco	*rbcL*
Self replication	Ribosomal RNAs	*rrn16* ^a^ *, rrn23* ^a^ *, rrn4.5* ^a^ *, rrn5* ^a^
	Transfer RNAs	*trnA-UGC* ^a,b^ *, trnC-GCA, trnD-GUC, trnE-UUC, trnF-GAA, trnG-GCC, trnH-GUG, trnI-CAU* ^a^ *, trnI-GAU* ^a,b^ *, trnK-UUU* ^b^ *, trnL-CAA* ^a^ *, trnL-UAA* ^b^ *, trnL-UAG, trnM-CAU, trnfM-CAU, trnN-GUU* ^a^ *, trnP-UGG, trnQ-UUG, trnR-UCU, trnR-ACG* ^a^ *, trnS-UGA, trnS-GGA, trnS-GCU, trnT-GGU, trnT-UGU, trnW-CCA, trnY-GUA, trnV-UAC* ^b^ *, trnV-GAC* ^a^
	Proteins of small ribosomal subunit	*rps2, rps3, rps4, rps7* ^a^ *, rps8, rps11, rps12* ^a,b^ *, rps14, rps15, rps16, rps18, rps19* ^a^
	Proteins of large ribosomal subunit	*rpl2* ^a,b^ *, rpl14, rpl16* ^b^ *, rpl20, rpl22, rpl23* ^a^ *, rpl32, rpl33, rpl36*
	Subunits of RNA polymerase	*rpoA, rpoB, rpoC1* ^b^ *, rpoC2*
Other genes	Acetyl-CoA carboxylase	*accD*
	Cytochrome c biogenesis	*ccsA*
	Envelope membrane protein	*cemA*
	Maturase	*matK*
	Protease	*clpP* ^b^
	Translation initiation factor	*infA*
Unknown	Conserved hypothetical chloroplast reading frames	*ycf1* ^a^ *, ycf2* ^a^ *, ycf3* ^b^ *, ycf4, ycf15* ^a^ *, ycf68* ^a^ *, lhbA*

^a^Genes located in the IR regions.

^b^Genes having introns.

### Comparison to the cp genomes from other Euphorbiaceae species

The size of the tung tree cp genome was found to be similar to those from Euphorbiaceae family species, *J. curcas*, *H. brasiliensis* and *M. esculenta* (Table 3). However, tung tree cp genome has the longest SSC region (18,758 bp), whereas *J. curcas* has the shortest SSC region (17,852 bp). Tung tree cp genome contains more genes (135) than other species, and among them 21 genes duplicated in IRs, while 16 genes duplicated in *M. esculenta*. As shown in Table 3, tung tree has the highest GC content (36.02%), while *J. curcas* has the lowest GC content (35.36%). Four conserved rRNAs were identified in every species. *J. curcas* and *H. brasiliensis* contain 78 coding genes, whereas *M. esculenta* has 79, and tung tree has 81 coding genes. Tung tree cp genome encodes 29 types of tRNAs, whereas *H. brasiliensis* and *M. esculenta* encode 30 (Table 3).

**Table 3:** Comparison of general features of Euphorbiaceae plastid genomes.

Genome feature	*Vernicia fordii*	*Jatropha curcas*	*Hevea brasiliensis*	*Manihot esculenta*
Total length (bp)	161528	163856	161191	161453
LSC length (bp)	89132	91756	89209	89295
SSC length (bp)	18758	17852	18362	18250
IR length (bp)	26819	27124	26810	26954
GC content (%)	36.02	35.36	35.74	35.87
Total genes	135	130	128	128
Genes duplicated in IR	21	17	19	16
rRNA gene duplicated in IR	4	4	4	4
Protein gene	81	78	78	79
tRNA gene	29	28	30	30
rRNA gene	4	4	4	4

### Repeat sequence analysis

The tung tree cp genome encloses 49 repeats with at least 21 base pairs (bp) per repeat unit (Table 4). These repeats include two complementary repeats, 21 direct (forward) repeats, 16 inverted (palindrome) repeats and 10 reverse repeats have 1,504 bp in length, which is about 0.93% of the genome. Most of these repeats are located in intergenic regions, while 8 repeats are located in the introns and in the protein-coding genes including *psaA*, *ycf1* and *ycf2*.

**Table 4:** Repeat sequences in the tung tree cp genome.

No.	Length (bp)	Repeat type	Repeat 1 start position	Repeat 2 start position	Repeat 1 location	Repeat 2 location
1	22	C	48854	78423	trnfM-CAU_trnS-UGA	trnfM-CAU_trnS-UGA
2	26	C	104466	146168	ycf15_trnV-GAC	ycf15_trnV-GAC
3	21	F	7396	146181	rpoA_psbN	rpoA_psbN
4	24	F	25372	25405	psbJ_atpB	psbJ_atpB
5	67	F	33717	33766	trnV-UAC_ndhC	trnV-UAC_ndhC
6	29	F	33738	33833	trnV-UAC_ndhC	trnV-UAC_ndhC
7	29	F	33787	33833	trnV-UAC_ndhC	trnV-UAC_ndhC
8	35	F	43695	45919	psaA	psaA
9	53	F	53710	75960	trnS-UGA_trnE-UUC	trnS-UGA_trnE-UUC
10	23	F	55290	55312	trnS-UGA_trnE-UUC	trnS-UGA_trnE-UUC
11	23	F	56751	56772	trnD-GUC_psbM	trnD-GUC_psbM
12	30	F	78078	78104	atpA_trnS-GCU	atpA_trnS-GCU
13	26	F	78159	78176	atpA_trnS-GCU	atpA_trnS-GCU
14	34	F	78333	78353	atpA_trnS-GCU	atpA_trnS-GCU
15	25	F	83781	83806	rps16_trnK-UUU	rps16_trnK-UUU
16	22	F	83784	83831	rps16_trnK-UUU	rps16_trnK-UUU
17	23	F	83809	83831	rps16_trnK-UUU	rps16_trnK-UUU
18	62	F	96620	96656	ycf2	ycf2
19	26	F	96620	96692	ycf2	ycf2
20	26	F	117126	117141	ycf1	ycf1
21	25	F	134591	134653	ndhF_trnN-GUU	ndhF_trnN-GUU
22	62	F	153942	153978	trnL-CAA_trnI-CAU	trnL-CAA_trnI-CAU
23	26	F	153942	154014	trnL-CAA_trnI-CAU	trnL-CAA_trnI-CAU
24	21	P	7396	104458	rpoA_psbN	rpoA_psbN
25	24	P	22678	22678	psbJ_atpB	psbJ_atpB
26	22	P	33682	33682	trnV-UAC_ndhC	trnV-UAC_ndhC
27	29	P	39744	80476	rps4_ycf3	rps4_ycf3
28	21	P	39749	49846	rps4_ycf3	rps4_ycf3
29	26	P	48023	48023	trnfM-CAU_trnS-UGA	trnfM-CAU_trnS-UGA
30	22	P	48862	79778	trnfM-CAU_trnS-UGA	trnfM-CAU_trnS-UGA
31	52	P	57302	57302	psbM_rpoB	psbM_rpoB
32	22	P	74732	74732	atpH_atpF	atpH_atpF
33	58	P	89003	89003	trnH-GUG_ycf2	trnH-GUG_ycf2
34	62	P	96620	153942	ycf2	ycf2
35	26	P	96620	153942	ycf2	ycf2
36	62	P	96656	153978	ycf2	ycf2
37	26	P	96692	154014	ycf2	ycf2
38	22	P	117973	117973	ycf1	ycf1
39	28	P	131410	131410	ndhD_ndhF	ndhD_ndhF
40	23	R	4544	4544	rpl36_rps11	rpl36_rps11
41	21	R	19798	19798	trnW-CCA_psbE	trnW-CCA_psbE
42	21	R	22672	22672	psbJ_atpB	psbJ_atpB
43	24	R	48637	48637	trnfM-CAU_trnS-UGA	trnfM-CAU_trnS-UGA
44	26	R	65276	65276	rpoC1	rpoC1
45	22	R	78009	78009	atpA_trnS-GCU	atpA_trnS-GCU
46	22	R	78029	78061	atpA_trnS-GCU	atpA_trnS-GCU
47	31	R	78030	78030	atpA_trnS-GCU	atpA_trnS-GCU
48	26	R	104466	104466	ycf15_trnV-GAC	ycf15_trnV-GAC
49	26	R	146168	146168	trnN-GUU_rps7	trnN-GUU_rps7

C: complement repeats, F: forward repeats, P: palindrome repeats, R: reverse repeats.

### Simple sequence repeat (SSR) analysis

81 SSR loci were identified, including 63 mononucleotide SSR loci (77.78%), five dinucleotide SSR loci (6.17%), and 13 other types of SSR loci (16.05%). Among them, there are 79 A or T repeats, one G repeat and one AG dinucleotide repeat. These SSR loci represent 0.937% of the complete cp sequence. 64 of the 81 SSR loci are located in intergenic regions, eight in gene-coding regions, six in intron regions, two between the gene-coding and intron regions, and only one between the intergenic regions and gene codingregions (see Supplementary Table S1).

### Variation analysis

**Figure 2:**
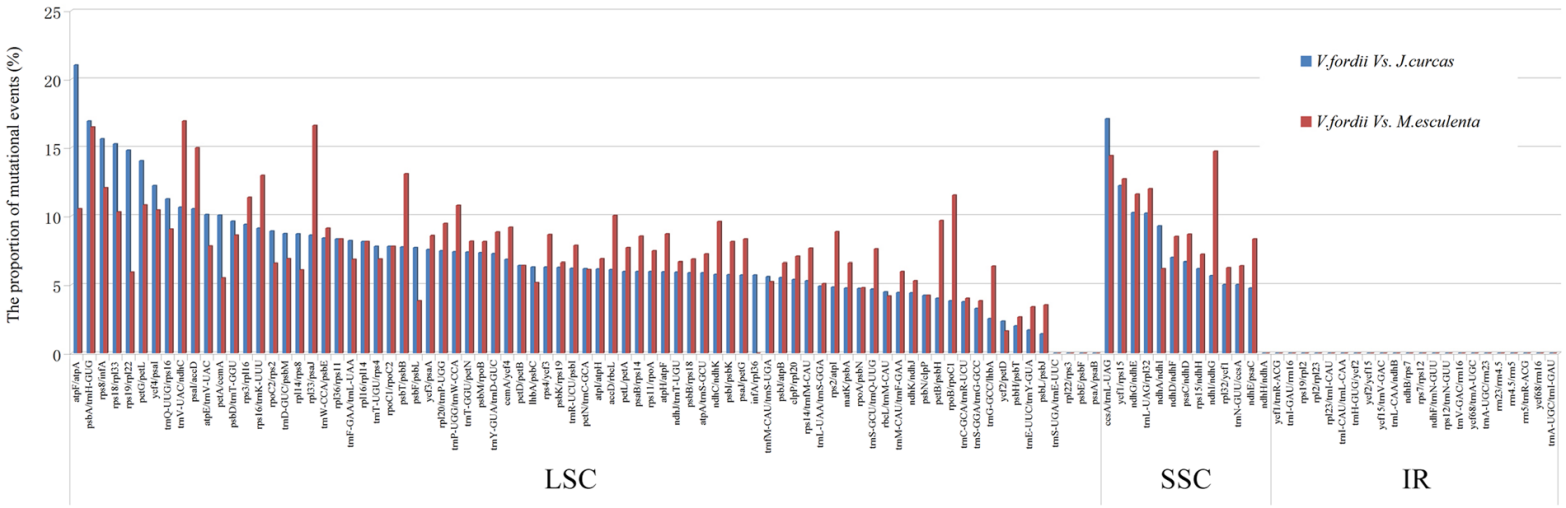
The variation analysis within intergenic spacer (IGS) regions between *V. fordii* and *J. curcas* or *M. esculenta*.

By comparing with the cp genome sequences from *V. fordii* and *J. curcas*, a total of 85 SNPs (single nucleotide polymorphisms) and 82 indels were identified. atpF/atpA is the most variable in the IGS within the LSC region (21.05% of variability). *V. fordii* and *M. esculenta* have identified 86 SNPs and 81 indels. Among them, 69 SNPs and 74 indels are within LSC region, 12 SNPs and seven indels are within SSC region. trnV-UAC/ndhC is the most variable in the IGS within the LSC region (16.96% of variability) (Fig. 2).

### IR contraction and expansion

**Figure 3:**
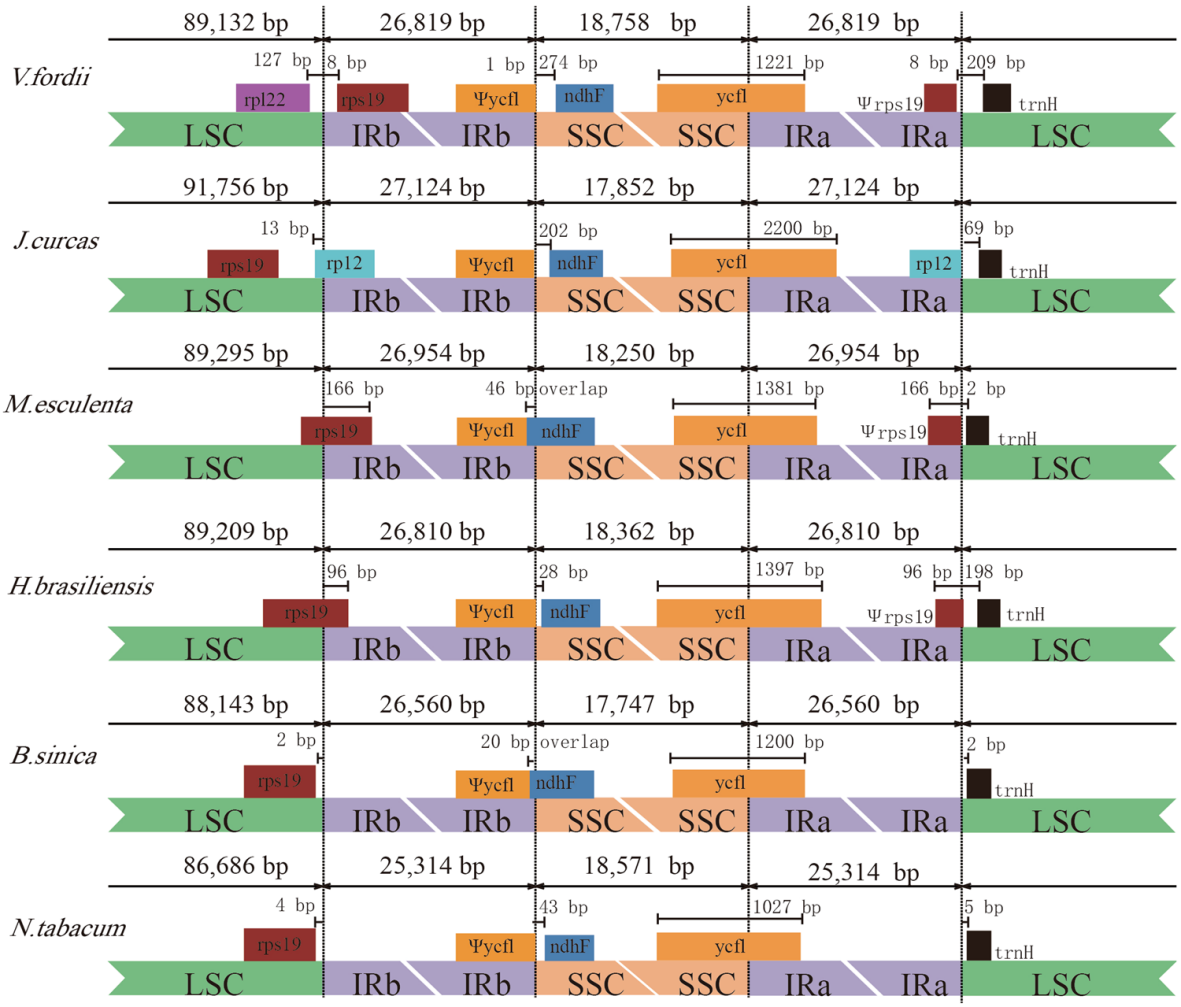
Comparison of the border regions of LSC, IR and SSC among six chloroplast genomes of basal eudicots.

Although IRs are the most conserved regions in cp DNA, the expansion and contraction at the borders of the IR regions are common evolutionary events, causing size variation of cp genomes^22, 23^. The IR-LSC and IR-SSC borders of tung tree cp genome were compared to those of the five basal eudicots (*J. curcas*, *M. esculenta*, *H. brasiliensis*, *B. sinica*, and *N. tabacum*). In all plant species, the IRb/SSC borders extend into the *ycf1* genes to create long *ycf1* pseudogenes with variable length. The length of *ycf1* pseudogene is 1,221 bp in tung tree, 2,200 bp in *J. curcas*, 1,397 bp in *H. brasiliensis* and 1,027 bp in *N. tabacum*. In addition, the *ycf1* pseudogene and the *ndhF* gene overlap *M. esculenta* and *B. sinica* cp genomes by 46 bp and 20 bp, respectively, but the *ndhF* genes of the other 4 species are all located in the SSC region, and it ranges from 274 bp from the IRb/LSC border in tung tree cp genome (Fig. 3). The *trnH-GIG* sequences are found in LSC regions of all cp genomes. This gene is 209 bp from the IRa/LSC border in tung tree cp genome. The *rps19* sequence is detected in the IR regions of tung tree cp genome and 8 bp apart from the LSC/IRb border, whereas this gene is located in the LSC in *J. curcas*, *B. sinica* and *N. tabacum*. In addition, the *rps19* gene is observed at the IRb/LSC border of two Euphorbiaceae plants, *M. esculenta* and *H. brasiliensis* (Fig. 3).

### Phylogenetic Analysis

**Figure 4:**
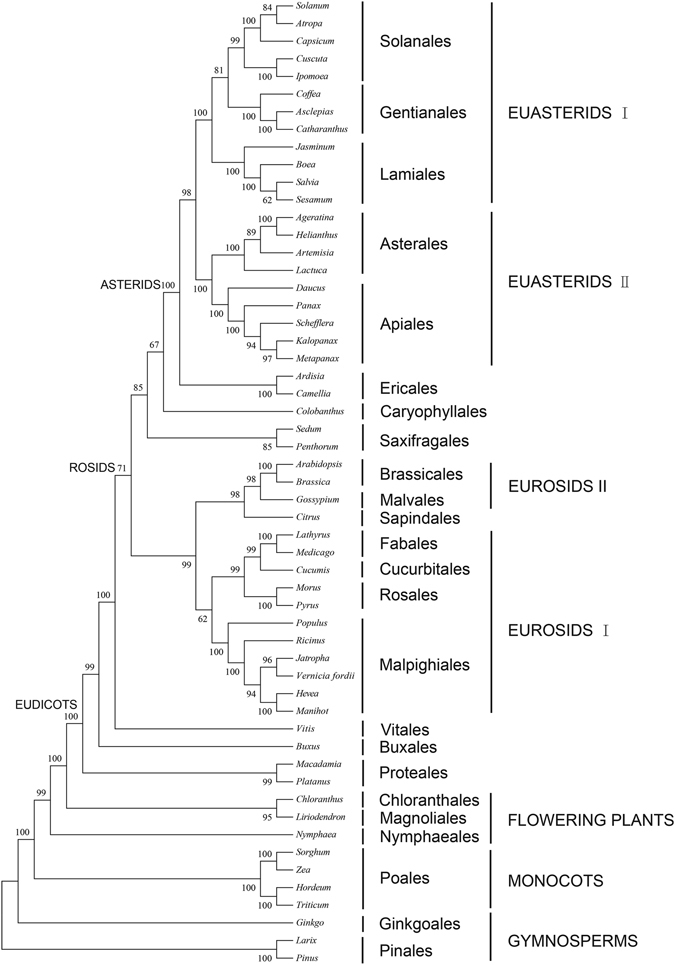
The maximum parsimony (MP) phylogenetic tree based on 36 protein-coding genes in the chloroplast genome. The numbers in each node was tested by bootstrap analysis with 1000 replicates.

To analyze the *V. fordii* phylogenetic position within asterid lineage, we aligned 55 complete cp genome sequences using the 36 protein-coding genes. The species representing 24 orders and included 3 outgroup taxa. The sequence analysis showed a fully resolved phylogenetic tree (12,995 in length of 0.51 for consistency index and 0.65 for retention index) (Fig. 4). The phylogenetic trees generated by ML and MP alignment have similar topologies (Figs 4 and S1). There are a total of 7,609 positions in the final dataset. *V. fordii* is placed as sister to *J. curcas* with a bootstrap (96). *V. fordii* is grouped to Malpighiales with *J. curcas*. There is a sister relationship among Falales, Cucurbitalesand Rosales.

## Discussion

The entire chloroplast genome of tung tree was determined using the third-generation sequencing (PacBio RS II System) method and assembled with the chloroplast genomes of the other Euphorbiaceae plants using the cp genomes of *J. curcas* and *M. esculenta* as references. The genome sequence was confirmed by Sanger sequencing of PCR-based products using specific primers (see Supplementary Table S2). As shown in Figure 1, the tung tree cp genome is a typical circle DNA, similar to those from Euphorbiaceae^7, 13, 24^.

Repeat sequences are useful for studying genome rearrangement and play an important role in phylogenetic analysis^25^. There are 49 repeats in the tung tree cp genome. A large number of repeats are distributed within IGS regions and the IRs account for the majority of repeats. In addition, we also find many repeats are present in the *ycf2* gene including two forward repeats and four palindrome repeats. The results are similar to those of previous studies on *Jatropha curcas* ^13^, *Citrus sinensis* ^16^ and Vitis^26^. Meanwhile, the non-coding regions in cp genomes are important for phylogenetic studies in angiosperms^27^. Most of the repeats are found in the non-coding regions of the tung tree cp genome.

In tung tree cp genome, 81 SSR loci with a length of at least 10 bp were identified (Table S1). All of the dinucleotides are composed of multiple copies of AT/TA repeats, and 75 of them are detected in the noncoding regions. These findings are similar to those of the other published results, i,e., repeats are typically found in the noncoding regions, especially in IGS regions of the cp genomes^17, 28, 29^. The SSRs in cp genomes was first reported in *Pinus radiata* ^30^. These SSRs can be useful biomarkers for genetic diversity.

The border regions of LSC-IRa, IRa-SSC, SSC-IRb and IRb-LSC represent highly variable regions with many nucleotide changes in cp genomes of closely related species. We compared the IR boundary regions of cp genome from six species in this study. The border of tung tree cp genome is differed slightly from that of other cp genomes. At the IRb and SSC border, the intergenic region of *ycf1* and *ndhF* in tung tree cp genome is larger (274 bp) than those in other species^13^. In addition, the SSC region in tung tree cp genome is also larger than those in other species. The long distance of IRb and SSC border could be a result of the expanding chloroplast genome of tung tree. The *rps19* gene of tung tree is entirely located in the IR regions, which is generally located in the LSC region or at the junction of LSC/IRb border in dicotyledons^31–33^. Previous studies have shown that *rps19* sequence is generally positioned in the IR regions of cp genomes from monocotyledon pineapple (*Ananas comosus*)^34^, and *Chionographis japonica* ^35^. Our results indicate that the *rps19* gene location is similar to monocotyledon. In Euphorbiaceae, though the IR region of tung tree cp genome is shorter than that of *J. curcas* and *M. esculenta*, it has more duplicated genes (21 genes) than those of *J. curcas* (17 genes) and *M. esculenta* (16 genes). The main reasons for these differences are that the *rps19* gene is duplicated in IR regions and that the *ycf15* and *ycf68* genes are found in tung tree; which are consistent with those results obtained from *Hevea brasiliensis* ^7^ and *Musa acuminata* ^36^. Meanwhile, *ycf15* and *ycf68* genes were identified as pseudogenes in tung tree, and *ycf68* sequence is found in the intron regions of trnI-GAU. The similar result has been reported in the cp genome sequence of *Pelargonium hortorum* ^15^.

It is reported that cp genomes in most land plants have two identical IR regions, which have lower the nucleotide substitution rates and fewer indels than LSC and SSC regions^37^. Similarly, few indels were identified in the IR regions of tung tree cp genome. IGSs and intron regions have more indels than protein-coding genes and thus evolve more quickly than protein-coding genes. Traditionally the nucleotide substitutions and indels in cp genomes have been used as DNA markers in the phylogenetic analysis of many land plants^38–40^.

In the Euphorbiaceae family several studies have analyzed the phylogenetic relationship based on chloroplast DNA sequences^7, 13, 24^. The phylogenetic evolution of *V. fordii* were studied here using 36 protein coding genes for 55 plant taxa (Supplementary Table S3), including 52 angiosperms and three outgroup gymnosperms (*Ginkgo*, *Larix* and *Pinus*). We used MP and ML analyses to construct an evolutionary tree involving 55 amino acid sequences. All 52 nodes were resolved well and reliable based on MP bootstrap value: 41 have strong bootstrap support of 95–100% and 11 have moderate support of 60–95%. *V. fordii* and the other four species in the family Euphorbiaceae are clustered into Malpighiales as a well-supported monophyly and placed within Eurosids I, which is similar to pervious work^41^. The phylegenetic tree indicates that subfamily Crotonoideae is a younger, more evolved group than subfamily Acalyphoideae (i.e. *Ricinus* in this study). However, the deep phylogeny within angiosperms differ from previous research in several ways^42, 43^. In our analysis, monocots forms a sister group to the remaining angiosperms, although it is often embedded in dicots in other studies. One possible reason is the heterogeneity between the nuclear and chloroplast genomes^44, 45^. There are a few disparities between the MP and ML trees in our analyses. This might be because maximum parsimony is sensitive to incongruent evolutionary rates at internal nodes^46^. In addition, *V. fordii* is suggested to be more closely related to *Jatropha* than to *Hevea* and *Manihot*.

## Conclusion

We presented the first complete nucleotide sequence of tung tree cp genome using PacBio RS II sequencing platforms. The tung tree cp genome (161,528 bp) was fully characterized and compared to the cp genomes of related species. We identified two inverted repeat regions and one small and one large single copy regions. The tung tree cp genome contained 114 unique genes coded for 81 proteins, four ribosomal RNAs and 29 transfer RNAs. Phylogenetic analysis suggests that *V. fordii* is a sister species of *J. curcas* within the Eurosids I. Our study provides vital molecular information for understanding of the cp genome of this commercially important woody oil tree.

## Material and Methods

### Plant materials and DNA sequencing

Tung tree leaves were obtained from a two years old self-bred progeny plant at Central South University of Forestry and Technology Germplasm Repository (CSUFTGR) (110° 29′ E, 28° 32′ N, Yong Shun, Ji Shou, Hunan, China). Based on the manufacturer’s instructions, the whole genomic DNA was extracted from 5 g of fresh leaves with DNeasy Plant Mini Kit (QIAGEN, CA, USA). After DNA was purified, 5 mg was used in library construction. In addition, a PacBio RS II platform^47^ was used for sequencing tung tree cp genome (Nextomics, Wu Han, China).

### Genome assembly and annotation

All sequenced reads were filtered through removing the adapter sequence and cutting off low quality bases in reads and assembled by HGAP 2.3.0 process^48^, Celera assembler (CA) assembled software^49^ and OLC assembly algorithm^50^. The cp genome was annotated using Dual Organellar GenoMe Annotator (DOGMA)^51^ and CPGAVAS (http://www.herbalgenomics.org/0506/cpgavas/analyzer/annotate). The predicted annotations were confirmed by BLAST^52^ search against the nucleotide database of NCBI (http://www.ncbi.nlm.nih.gov/gorf). Uncertain annotations for protein-coding sequences, tRNAs and mRNAs genes were corrected after being compared with near edge species.

### Genome Validation

Because chloroplast genomes exhibit a greater degree of conservation in most of the plants, we compared the complete cp genome sequences among tung tree, *Jatropha* [NC_012224], and *Manihot* [EU117376] in NCBI plastid database. The sequence discrepancies between tung tree and *Jatropha* or *Manihot* cp genome sequences were validated by PCR amplification and Sanger sequencing. Ten different bases between IRa and IRb regions were also amplified by PCR. PCR were used to verify differences in the sequence of the preliminary cp genome assembly using 29 pairs of forward and reverse primers (see Supplementary Table S2).

### Analysis of cp genome sequence

GenomeVx software^53^ was used to draw the circular map of the tung tree chloroplast genome. Mauve software^12^ and mVISTA program were applied to identify similarities among different cp genomes (http://genome.lbl.gov/vista/mvista/submit.shtml)^54^. REPuter^55^ was utilized to identify forward (direct) repeats, reverse sequences, complementary and palindromic sequences with at least 21 bp in length and 90% of sequence identity. The distributions of simple sequence repeats (SSRs) were predicted using the microsatellite search tool MISA^56^. Insertions and deletions (indels), as well as nucleotide substitutions and inversions were scored as single independent characters. The formula (*NS* + *ID*)/*L* × 100 (NS, nucleotide substitutions number; ID, indels number; L, the aligned sequence length) was used to calculate the ratio of mutation events. In addition, the contraction/expansion regions of the inverted repeat (IR) were compared among *V. fordii*, *J. curcas*, *M. esculenta*, *H. brasiliensis*, *B. sinica*, and *N. tabacum*.

### Phylogenetic analysis

Fifty-two angiosperm and three gymnosperm taxa typically possess a set of 36 protein-coding genes: atpA, atpB, atpE, atpH, atpI, petA, petB, petD, petG, petN, psaA, psaB, psaJ, psbA, psbC, psbD, psbF, psbH, psbJ, psbK, psbM, psbN, psbT, matK, rbcL, rpl33, rpoA, rpoB, rps2, rps3, rps4, rps8, rps18, rps11, rps14, and ccsA. These genes are present in all 55 cp genomes published in the NCBI database (see Supplementary Table S3). The maximum parsimony (MP) and maximum likelihood (ML) were performed to infer the evolutionary relationship. MUSCLE^57^ was used to align sequences followed by manual adjustment. MEGA*6.0^58^ was used for MP analysis using a heuristic search selected. Bootstrap analysis was done with 1,000 replicates with TBR branch swapping. ML analysis was conducted using FastTree v2.1.3^59, 60^ with the default parameters. The nucleotide substitution model we chose was GTRGAMMA model, which was the common model reported in the literature. The 1000 replications were used to calculate local bootstrap probability of each branch.

## Electronic supplementary material

Supplementary Figure S1

Supplementary Dataset 1

Supplementary Dataset 2

Supplementary Dataset 3
