# Supplementary material for: The complete chloroplast genome sequence of tung tree (Vernicia fordii): Organization and phylogenetic relationships with other angiosperms
Source: Sci Rep. 2017 May 12;7:1869. doi: 10.1038/s41598-017-02076-6 (PMC5431841; doi:10.1038/s41598-017-02076-6)
Supplement: Supplementary file 1 — Supplementary Figure S1 [file 41598_2017_2076_MOESM1_ESM.pdf]

The complete chloroplast genome sequence of tung tree (*Vernicia fordii*): Organization and phylogenetic relationships with other angiosperms

Ze Li[1,2], Hongxu Long[1,2], Lin Zhang[1,2], Zhiming Liu[1,2,3], Heping Cao[4], Mingwang Shi[5,*] & Xiaofeng Tan[1,2,*]

[1] Key Laboratory of Cultivation and Protection for Non-Wood Forest Trees, Ministry of Education, Central South University of Forestry and Technology, Changsha, Hunan 410004, China
[2] Cooperative Innovation Center of Cultivation and Utilization for Non-Wood Forest Trees of Hunan Province, Central South University of Forestry and Technology, Changsha, Hunan 410004, China
[3] Department of Biology, Eastern New Mexico University, Portales, New Mexico 88130, USA
[4] U.S. Department of Agriculture, Agricultural Research Service, Southern Regional Research Center, New Orleans, Louisiana 70124, USA
[5] Henan Institute of Science and Technology, Xinxiang, Henan 453003, China

Correspondence and requests for materials should be addressed to Xiaofeng Tan and Mingwang Shi (email: tanxiaofengcn@126.com; shimw888@126.com)
Tel: +86-0731-85623416; Fax: +86-0731-85623416
Whose present postal address: 498 South Shaoshan Road, Changsha, Hunan 410004, China

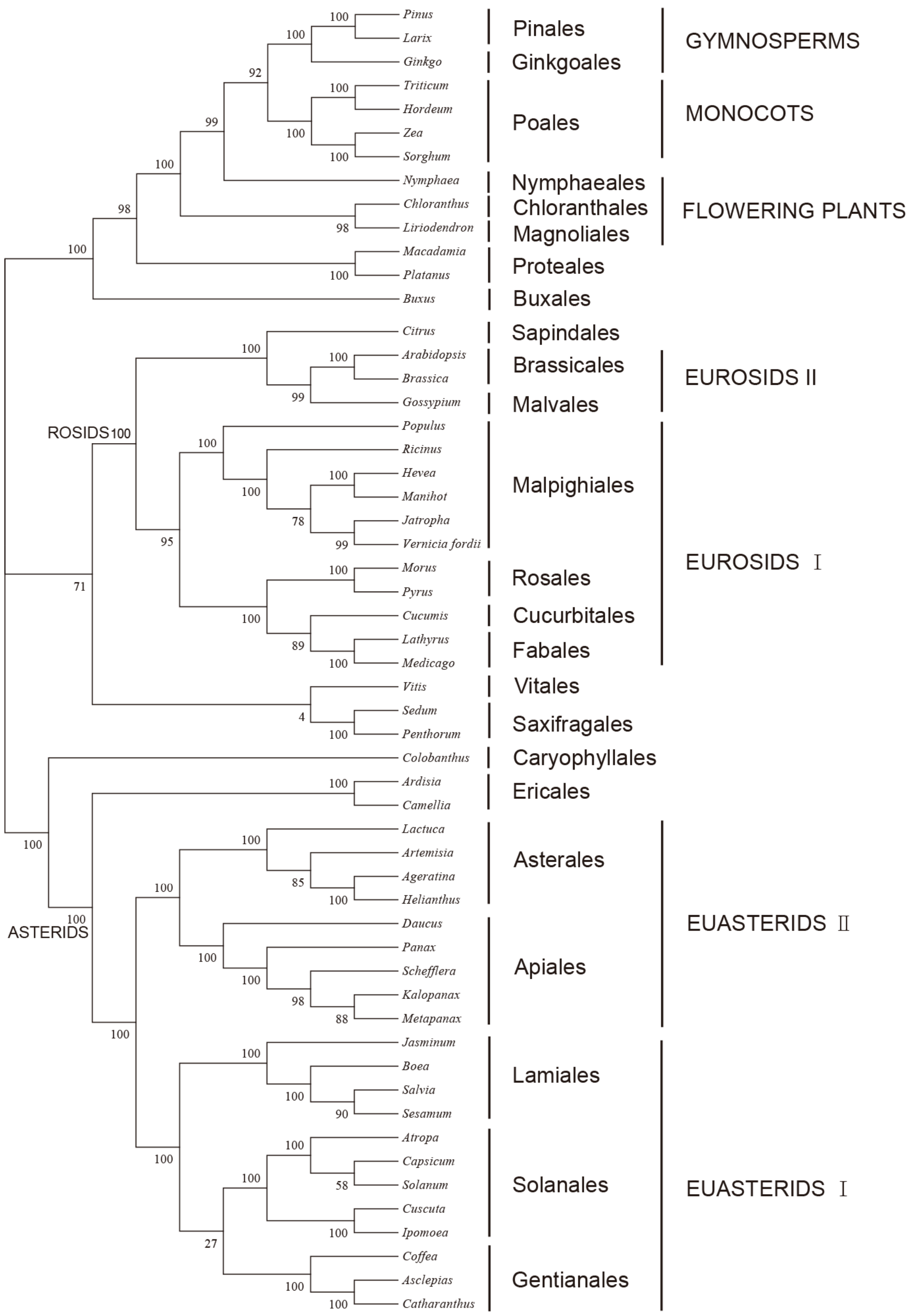

Figure S1. The maximum parsimony (MP) phylogenetic tree based on 36 protein-coding genes in the chloroplast genome. The numbers in each node was tested by bootstrap analysis with 1000 replicates
